# Effect of subcutaneous needling on visual analogue scale, IgG and IgM in patients with lumbar disc herniation: Study protocol clinical trial (SPIRIT Compliant)

**DOI:** 10.1097/MD.0000000000019280

**Published:** 2020-02-28

**Authors:** Jiangxia Yang, Chen Yang, Yajie Wang, Ning Li, Xingzhang Yao, Bowen Yang, Xia Xu, Xingyong Li

**Affiliations:** a Gansu Provincial Hospital of Traditional Chinese Medicine; b Gansu University of Chinese Medicine; c Gansu Provincial People's Hospital, Lanzhou, Gansu, China.

**Keywords:** acupuncture, lumbar disc herniation, protocol

## Abstract

**Background:**

Lumbar disc herniation (LDH) is a disease commonly seen in clinical practice. In the majority of such patients presenting in clinic, the symptoms can be relieved or even abolished after non-surgical treatment. Floating needle therapy has attracted considerable attention as a promising non-surgical technique to treat LDH, as demonstrated in previous studies. The purpose of the present study was to evaluate the outcomes of patients treated using this therapy in a single blind and randomized controlled trial by recording patient report questionnaires and objective test data, and to explore the feasibility and preliminary effects of floating needle therapy for patients with LDH.

**Methods:**

A total of 80 patients who fulfilled the inclusion criteria were randomly divided into a Fu's subcutaneous needling (FSN) group and an acupuncture group then treated in accordance with procedures appropriate for a single blind and randomized controlled trial. The FSN group received 12 FSN therapy sessions over a 3-week period, and the acupuncture group received acupuncture therapy at specified points using acupuncture needles. The principal measurements were scored using the visual analogue scale (VAS), Japanese Orthopedic Association (JOA) Score, and Oswestry disability index (ODI) before and 3 weeks after treatment. Secondary measurements included immune function IgG and IgM measurements performed at the same time and adverse reactions during treatment.

**Results:**

The results of this trial will be published on the website of China Clinical Trial Registration Center (http://www.chictr.org.cn/searchprojen.aspx) and in peer-reviewed journals or academic conferences.

**Conclusions:**

This study will explore the feasibility and preliminary effects of floating needle therapy for the treatment of patients with LDH.

**Registration:**

PROSPERO (registration number ChiCTR1900024045).

## Abstract 2

Supplemental Digital Content is available in the text

## 1 Introduction

**Figure 1:**
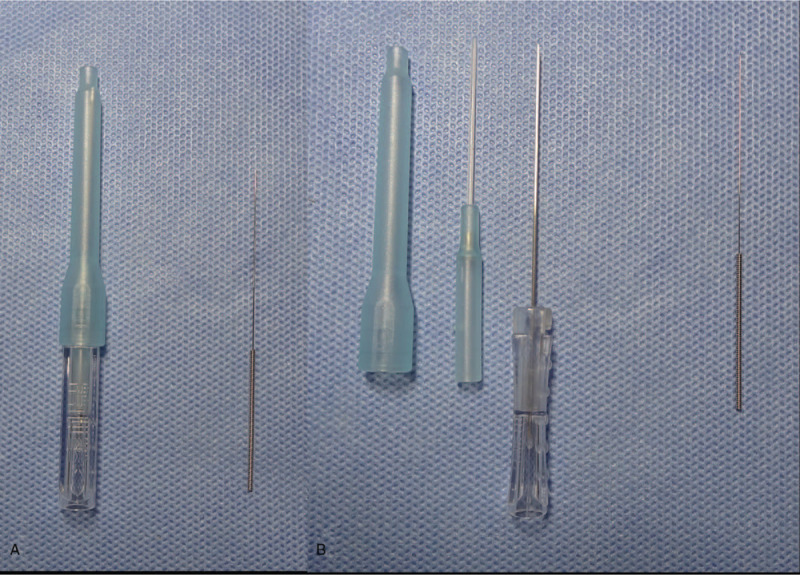
The difference between FSN and acupuncture. (A) Undismantled FSN and acupuncture (right 1). (B) Demolition of FSN and acupuncture (right 1). FSN = Fu's subcutaneous needling.

Lumbar disc herniation (LDH) results from a pathological change, with lumbar leg pain as the principal symptom, caused by the degeneration of a lumbar intervertebral disc, partial or complete rupture of the annulus fibrosus because of applied external force, outward protrusion of the nucleus pulposus, and cartilage endplate alone or together with stimulation or compression of the nerve root.^[1–3]^ Approximately 9% of the individuals globally have suffered from LDH, and as the population ages, this proportion is likely to increase.^[4]^ The cost of surgical treatment of LDH is approximately 27,000 US dollars, but there are multiple adverse reactions and complications.^[5]^ This factor deters many patients. Clinical experiments^[6–10]^ indicate that acupuncture and Fu's subcutaneous needling (FSN), and especially the latter (Fig. 1) represent the potential treatment for LDH. FSN consists of the needle holder, hose sleeve, and solid stainless steel needle core with sufficient hardness. FSN therapies are a combination of “Traditional Chinese acupuncture medicine” and “modern medicine”. Its therapeutic target is the tender point, namely the myofascial trigger point^[11]^ (MTrP), a key factor that causes pain in more than 93% of patients and the only cause of pain in 85%.^[12,13]^ An FSN therapy is a method of sweeping and dispersing using disposable FSN needles to swing left and right after horizontally inserting the needle around an MTrP or the subcutaneous tissue of adjacent limbs, thus achieving analgesia. The purpose of this study was to explore the feasibility and preliminary results of using floating needle therapy for LDH patients.

## 2 Methods

### 2.1 Study design

The present study is a single-blind, randomized controlled clinical study that applies the ethical principles of China's Ministry of Health's “Ethical Review Measures for Biomedical Research Involving Human Beings (Trial 2007)”, “Regulations on Clinical Trials of Medical Devices (2004)”, WMA's “Helsinki Declaration”, and CIOMS's “International Ethical Guidelines for Biomedical Research on Human Beings”. The project was evaluated and approved by the Research Ethics Committee of Gansu Provincial Hospital of Traditional Chinese Medicine (Ethical batch No. FJ/01-IRB/C/018-V3.0, Ethics Review Adoption Form places in the Supplemental Digital Content). The protocols were first released and last updated in June 2019. Treatment will be conducted in Gansu Provincial Hospital of Traditional Chinese Medicine from January 1, 2020 to June 30, 2020. The schedule of pre-intervention, intervention, and evaluation procedures will be presented in Supplemental material 1. A flow chart representing the stages of this study is shown in Supplemental material 2.

### 2.2 Participants

The following eligibility criteria apply to all patients for recruitment into the study. Patients will be recruited through the acupuncture clinic of Gansu Provincial Hospital of Traditional Chinese Medicine. After oral explanation and reading of the procedures used in the study, patients agreeing to participate will sign an informed consent form.

Inclusion criteria:

- Aged between 20 and 75 years;
- Fulfilling the diagnostic criteria for LDH published by the North American Spinal Surgery Association^[14]^ in 2013 (manual muscle testing, sensory testing, supine straight leg raise test, Lasegue sign and crossed Lasegue sign are all considered diagnosable);
- Those unconcerned about FSN or acupuncture treatment;
- Willing to enter the clinical trial and provide signed informed consent.

Exclusion criteria:

- Compression fracture or tuberculosis found at the lesion site;
- Horsetail nerve damage syndrome leading to saddle paralysis, defecation, weakness of micturition, urinary retention and urinary incontinence, and absolute surgical indications;
- Skin infection, ulcers, scars, or tumors that cannot be punctured;
- Coagulation dysfunction;
- Severe arrhythmia, heart failure, chronic obstructive pulmonary disease, an endocrine disease, epilepsy, or mental illness;
- Patients with infectious diseases, acute inflammation, fever, or pregnant women;
- Combined medication or participation in other trials during treatment.

### 2.3 Study interventions

#### 2.3.1 FSN group

*FSN selection*: Disposable floating needles (medium: M) produced by Nanjing Paifu Medical Science and Technology Co., Ltd. were selected for use in this trial. These have passed inspection and have obtained a production license (License no: su Shi Yao Jian Xie Xu 20040104; Registration no: 20152270832).

*Procedure*: The patient is placed in a prone position and the MTrP P1, P2, P3, and P4 ascertained (the four positions determined according to positions causing pain, where P1 and P2 points are at the waist, P3 points at the thigh and P4 points on the calf). The position of needle insertion is 3 to 5 cm below or outside the MTrP. An FSN needle insertion device is required to insert the needle. Insertion of the needle tip is required to be at an oblique angle of 15° to 20° relative to the surface of the skin. The preferable insertion depth of the needle is just beyond the depth of the subcutaneous muscle layer. The thumb, index finger, and middle finger are used to move the upper part of the needle body, lift and pinch the needle handle, and sense the tightness of the muscle as the needle tip moves in response to movement of the fingers. The needle body is then gently and slowly lifted until the needle tip leaves the muscle layer and enters the subcutaneous layer for additional sweeping treatment. The duration of scanning time at each needle entry point is approximately 2 minutes, each position scanned approximately 200 times. The treatment cycle requires 3 weeks, usually once a day for the first 3 days, then 2 days apart, for a total of 12 occasions.

#### 2.3.2 Acupuncture group

*Acupuncture needle selection*: Jiajian brand disposable sterile Acupuncture needles, 0.30 × 40 mm and 0.30 × 75 mm, with a production license no: 20060095. *Procedure*: Acupuncture of the bilateral Shenshu (BL 23), Jiaji (BX-B2), Weizhong (BL 40), and Ashi acupuncture points (irregular). The treatment cycle selected for this study was 3 weeks, usually once per day for the first 3 days, then 2 days apart, for a total of 12 occasions.

### 2.4 Sample size calculation

In order to determine the number of participants required for each experimental group, the variability of results in a previously published article^[7]^ was used in the calculation. The main results of the two studies were obtained under similar circumstances. The sample size required for an unpaired *t* test was 60 individuals, including 32 in the FSN group and 28 in the acupuncture group. The calculation was performed using a significance level of .05 (i.e., 5% of type I errors, resulting in a 95% confidence interval) and an absolute error of 5%. Considering a dropout rate of 10%, 80 patients are required to generate 80% of the power, at a significance level of .05.^[15]^

### 2.5 Randomization and allocation concealment

In the present study, the 80 recruited patients were divided into FSN and acupuncture groups at a ratio of 1:1. A statistical expert not involved in the design of the study used R Package v3.4.4 to generate a set of random sequences, to be sealed in opaque envelopes prepared in advance. Only FSN and acupuncture practitioners were authorized to open the envelopes to obtain each grouping code.

### 2.6 Single-blinding

FSN and acupuncture practitioners were not provided details of treatment plans or grouping information within the envelope in advance. No participant or relevant medical personnel was authorized to interfere with individual treatment selections. Evaluators are blinded to the treatment each patient has received, their function being to assist each patient in completion of the various questionnaires and scoring the respective scales. It is intended that the independent biostatisticians are also blinded when conducting the statistical analysis.

### 2.7 Discontinuation criteria

- Any serious adverse reactions or other acute or severe event during the study, preventing continued participation in the study;
- Subjects requesting to withdraw from the study midway;
- Patients not cooperating with the treatment plan, preventing valid treatment execution even after repeated explanation by the clinician.
- Results of those exceeding half of the total course of treatment available for inclusion in the statistical analysis of treatments.

### 2.8 Outcomes

#### 2.8.1 Primary outcomes

##### 2.8.1.1 VAS^[16]^

Patients were marked on a 100-mm visual analogue scale prior to and 3 weeks following treatment for assessment of their pain level (from no pain to very severe), which was then recorded by the evaluator.

##### 2.8.1.2 JOA Score^[17]^

Clinical symptoms and difficulty in performing tasks of daily living were evaluated according to the therapeutic effects evaluation standard of the Japanese Orthopedics Association (JOA) for lumbago. All scores before and 3 weeks after treatment were recorded and total scores calculated, in accordance with JOA's score for lumbago and leg pain severity.

##### 2.8.1.3 **ODI**^[18]^

Prior to and 3 weeks following treatment, the Oswestry disability index (ODI) of each patient was recorded, principally referring to pain (degree of pain, impact of pain on sleep), single functions (lifting/carrying, sitting, standing, walking) and individual comprehensive functions (daily self-care capability, social activities, travel).

#### 2.8.2 Secondary outcomes

Autoimmune theory has been proposed as a factor responsible for immune events due to the special anatomical structure of the intervertebral disc. The existence of the nucleus pulposus as an isolation antigen, once exposed to the immune system, may be recognized as a non-self component, leading to stimulation of an immune response resulting in chronic inflammation, eventually resulting in lumbar and leg pain.^[19–21]^ Furthermore, studies have established that the elevated levels of IgG and IgM in serum are positively correlated with the severity of lumbago and leg pain.^[22]^ Therefore, IgG and IgM immune function measurements before and 3 weeks after treatment are important parameters for detection of treatment effects and adverse reactions during treatment and will be recorded.

### 2.9 Complications and adverse events during and after treatment

All expected and unexpected adverse events will be recorded during practitioner contact time throughout the study. Adverse events related to FSN and acupuncture are commonly hematoma and syncope during treatment. All serious adverse events will be immediately reported to the study contact in order to further investigate the cause.

### 2.10 Data management

After data collection is completed, all data will be recorded independently by two trained research assistants using paper case report forms (CRFs). Since, acupuncture and FSN are known to have minimal risk, a formal data monitoring committee is not required. Independent investigators employed by the hospital will regularly monitor and review the experimental data.

### 2.11 Statistical analysis

Since FSN and acupuncture are known to have only minimal risk, implementation of additional safety precautions is not required. Sensitivity analysis will be conducted to determine the impact of incomplete records on the results. Missing data will not be estimated. Statistical analysis will be performed using R package v3.4.4 software. If a Shapiro–Wilk test indicates that the data are normally distributed, a *t* test is appropriate for calculation and comparison of differences between two groups, otherwise a Mann–Whitney *U* test is suitable. A *χ*^2^ or Fisher exact test is relevant for calculation of differences in count data.

### 2.12 Ethics and dissemination

Patients and the public were not involved in the design of this study. The participants will be informed of the result of this study during the follow-up visit. Besides, we will enlist their help in disseminating the research findings.

## 3 Results

The results of this trial will be published on the website of the China Clinical Trial Registration Center and in peer-reviewed journals or academic conferences.

## 4 Discussion

It has been established that conservative treatment, especially FSN, is effective in relieving LDH pain symptoms and promoting functional recovery. In the era of opioid abuse and excessive use of non-steroidal anti-inflammatory drugs to relieve lumbago and leg pain symptoms, floating needle therapy has good prospects for the non-surgical relief of such patient incapacity. At present, the theory explaining the analgesic mechanisms of floating needle treatments are thought to mainly involve loose connective tissues^[23]^ with special structures representing the material basis for its effectiveness. Subcutaneous loose connective tissues in a liquid crystal state exhibit piezoelectric and anti-piezoelectric effects. When floating needles are used, especially when performing a sweeping action, the spatial configuration of the loose connective tissue in its liquid crystal state changes and bioelectricity is released. Loose connective tissue has excellent conductivity and can conduct bioelectricity with high efficiency. When the bioelectricity reaches an MTrP, it produces an anti-piezoelectric effect, changes cellular ion channel, mobilizes the internal disease resistance mechanisms, thus relieving pain rapidly. There is also a doubtful nerve theory,^[24]^ which suggests that FSN therapeutics stimulate pain-transmitting nerves, which can block the conduction of pain-sensing fibers in those nerves and inhibit the nociceptive stimulation reaction of spinal dorsal horn cells, thus achieving an analgesic effect.

There are a number of limitations to the present study. Firstly, there may be differences in patient gender, age, lumbar spine segments manifesting disease, prominence, etc, so sub-group analysis may be required to judge the effectiveness of FSN for different sub-groups. Secondly, because VAS, JOA Score, ODI, and other indicators are subjective, the educational level of patients and their cognitive ability may influence the results, biasing the outcome indicators. Finally, this study was conducted in China, and the subjects were mostly Chinese. Whether FSN is effective for other races requires confirmation using a multi-center study in the future.

## Author contributions

**Data collection and collation:** Chen Yang and Ning Li

**Funding acquisition:** Jiangxia Yang

**Project administration:** Jiangxia Yang and Xingyong Li

**Protocol draft:** Jiangxia Yang and Chen Yang

**Study design:** Chen Yang, Xingyong Li and Xia Xu

**Therapists:** Xingzhang Yao, Bowen Yang, Yajie Wang

**Validation:** Xingyong Li and Xia Xu

**Supervision:** Xingyong Li.

**Validation:** Xingyong Li.

**Writing – review & editing:** Xingyong Li.

## Supplementary Material

Supplemental Digital Content

## Supplementary Material

Supplemental Digital Content

## Supplementary Material

Supplemental Digital Content
