# Supplementary material for: Effect of subcutaneous needling on visual analogue scale, IgG and IgM in patients with lumbar disc herniation: Study protocol clinical trial (SPIRIT Compliant)
Source: Medicine (Baltimore). 2020 Feb 28;99(9):e19280. doi: 10.1097/MD.0000000000019280 (PMC7478818; doi:10.1097/MD.0000000000019280)
Supplement: Supplemental Digital Content [file medi-99-e19280-s001.pdf]

附件 1 伦理审查意见（FJ/01-IRB/C/018-V3.0）

伦理审查意见的通知

意见号	2019-018-01		
项目名称	浮针对腰椎间盘突出症患者 VAS 及 IgG、IgM 的影响		
项目类别	甘肃省中医药管理局		
研究单位	甘肃省中医院		
主要研究者	杨江霞		
审查类别	专家论证	审查方式	快速审查
审查日期	2019.3.12	审查地点	
审查委员	盛丽 吴心音		
审查文件	科研项目伦理审查申请表；主要研究者简历；研究经济利益声明；申请书；研究方案（1.0 版）；知情同意书（1.0 版）。		

审查意见

根据卫生部《涉及人的生物医学研究伦理审查办法（试行 2007）》、SFDA《药物临床试验质量管理规范（2003）》、《医疗器械临床试验规定（2004）》、WMA《赫尔辛基宣言》和 CIOMS《人体生物医学研究国际道德指南》的伦理原则，经本伦理委员会审查，意见如下：

同意申报。

主任委员签字	[signature]
伦理委员会	甘肃省中医院伦理委员会（盖章）
日期	2019.3.12

甘肃省中医院伦理委员会
