# Supplementary material for: Effect of subcutaneous needling on visual analogue scale, IgG and IgM in patients with lumbar disc herniation: Study protocol clinical trial (SPIRIT Compliant)
Source: Medicine (Baltimore). 2020 Feb 28;99(9):e19280. doi: 10.1097/MD.0000000000019280 (PMC7478818; doi:10.1097/MD.0000000000019280)
Supplement: Supplemental Digital Content [file medi-99-e19280-s002.doc]

| The schedule of trial enrolment,interventions and assessments | | | |
| --- | --- | --- | --- |
|  | **enrolment** | **Intervention period** | |
|  | **pre-interventiont** | **intervention** | **evaluation procedures** |
| **Enrolment** |  |  |  |
| **Informed consent** | **•** |  |  |
| **Assessment of eligibility** | **•** |  |  |
| **Randomisation** | **•** |  |  |
| **Interventions** |  |  |  |
| **FSN** |  | **•** |  |
| **Acupuncture** |  | **•** |  |
| **Assessments** |  |  |  |
| **VAS** | **•** |  | **•** |
| **JOA** | **•** |  | **•** |
| **ODI** | **•** |  | **•** |
| **IgG** | **•** |  | **•** |
| **IgM** | **•** |  | **•** |
| **Postoperative complications and adverse events** |  | **•** | **•** |

FSN，Fu's subcutaneous needling；VAS，Visualanalogue scale； JOA，Japanese Orthopedic Association；ODI,

Oswestry disability index.
