# Supplementary material for: Effect of subcutaneous needling on visual analogue scale, IgG and IgM in patients with lumbar disc herniation: Study protocol clinical trial (SPIRIT Compliant)
Source: Medicine (Baltimore). 2020 Feb 28;99(9):e19280. doi: 10.1097/MD.0000000000019280 (PMC7478818; doi:10.1097/MD.0000000000019280)
Supplement: Supplemental Digital Content [file medi-99-e19280-s003.pptx]

## Slide 1
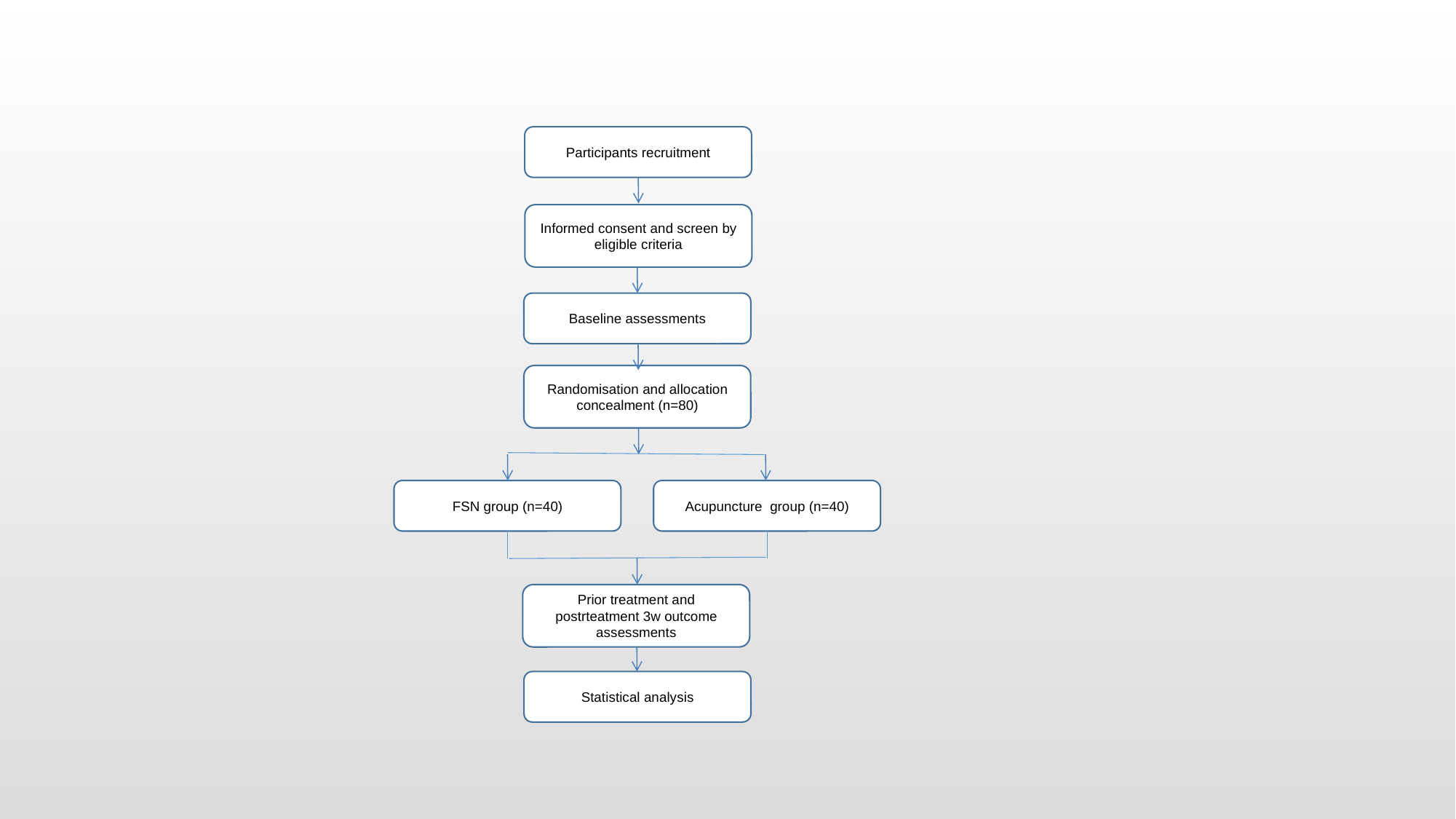

Participants recruitment
Informed consent and screen by eligible criteria
Baseline assessments
Randomisation and allocation concealment (n=80)
FSN group (n=40)
Acupuncture group (n=40)
Prior treatment and postrteatment 3w outcome assessments
Statistical analysis
